# TNR and conservation on a university campus: a political ecological perspective

**DOI:** 10.7717/peerj.312

**Published:** 2014-03-18

**Authors:** Jonathan Dombrosky, Steve Wolverton

**Affiliations:** Department of Geography, University of North Texas, Denton, Texas, USA

**Keywords:** TNR, Political ecology, Small animal conservation, Free-ranging cats, Domestication, Ethnobiology

## Abstract

How to manage the impact of free-ranging cats on native wildlife is a polarizing issue. Conservation biologists largely support domestic cat euthanasia to mitigate impacts of free-ranging cat predation on small animal populations. Above all else, animal welfare activists support the humane treatment of free-ranging cats, objecting to euthanasia. Clearly, this issue of how to control free-ranging cat predation on small animals is value laden, and both positions must be considered and comprehended to promote effective conservation. Here, two gaps in the free-ranging cat—small-animal conservation literature are addressed. First, the importance of understanding the processes of domestication and evolution and how each relates to felid behavioral ecology is discussed. The leading hypothesis to explain domestication of wildcats (*Felis silvestris*) relates to their behavioral ecology as a solitary predator, which made them suited for pest control in early agricultural villages of the Old World. The relationship humans once had with cats, however, has changed because today domesticated cats are usually household pets. As a result, concerns of conservation biologists may relate to cats as predators, but cat welfare proponents come from the position of assuming responsibility for free-ranging household pets (and their feral offspring). Thus, the perceptions of pet owners and other members of the general public provide an important context that frames the relationship between free-ranging cats and small animal conservation. The second part of this paper assesses the effects of an information-based conservation approach on shifting student’s perception of a local Trap–Neuter–Return (TNR) program in introductory core science classes at the University of North Texas (UNT). UNT students are (knowingly or unknowingly) regularly in close proximity to a TNR program on campus that supports cat houses and feeding stations. A survey design implementing a tailored-information approach was used to communicate what TNR programs are, their goals, and the “conservationist” view of TNR programs. We gauged favorability of student responses to the goals of TNR programs prior to and after exposure to tailored information on conservation concerns related to free-ranging cats. Although these results are from a preliminary study, we suggest that an information-based approach may only be marginally effective at shifting perceptions about the conservation implications of free-ranging cats. Our position is that small animal conservation in Western societies occurs in the context of pet ownership, thus broader approaches that promote ecological understanding via environmental education are more likely to be successful than information-based approaches.

## Introduction

Values, decisions, attitudes, and behaviors concerning free-ranging cat populations and conservation biology in the United States are diverse and polarized (Robertson, 2008). The study of the converging effects of social, economic, and political factors that influence perceptions and behaviors regarding environmental issues is referred to as political ecology (Robbins, 2012). Feral cats (*Felis silvestris catus*) are those that have been released or have escaped their owners and/or are the offspring of cats that live in the wild (i.e., feral cats are not pets). Free-ranging cats are those that roam freely outdoors without supervision; here we use the term “free-ranging” to refer to feral cats and roaming pet cats. One way that the relationship between free-ranging cats and people has developed is through establishment of ‘trap, neuter, and return’ (TNR) programs. These programs strive to minimize free-ranging cat populations through live-trapping, veterinary sterilization, and subsequent release to the areas where cats were trapped. Avoidance of euthanasia is crucial for TNR advocates. In contrast, control of cat populations is important for conservation biologists, who argue that TNR does not curtail, and in fact enables, predation on small wild animals. TNR programs are controversial because conservationists and TNR supporters hold differing attitudes and beliefs, which lead to contrasting decisions and behaviors, placing the ‘free-ranging cat debate’ squarely in the realm of political ecology.

Conservation biologists have demonstrated that free-ranging cats are efficient predators of small wild animals, such as native song birds, small mammals, reptiles, and amphibians (Coman & Brunner, 1972; Woods, McDonald & Harris, 2003; Hawkins, Grant & Longnecker, 2004; Balogh, Ryder & Marra, 2011; Loss, Will & Marra, 2013). There is an ethical concern among conservationists that can be summarized as “the presence of domestic cats on landscapes is a function of human behavior (ownership and neglect of domestic cats). The predatory behavior of free-ranging cats combined with their abundance wreaks havoc on wild animal populations. Therefore, society has an obligation to solve this problem”. In the minds of many conservation biologists, this ethical position is absolute (Longcore, Rich & Sullivan, 2009; Lepczyk et al., 2010; Dauphiné & Cooper, 2011). Although other concerns surround TNR programs, such as high disease loads in cats (Dabritz et al., 2006; Jessup & Hutchins, 2013; Mathusa, 2013), the central concern is the effect of free-ranging cat predation on small animals. TNR proponents adopt a different position; their first concern is for the welfare of free-ranging cats as living beings. Their position is, “humans have adopted cats, have a responsibility to ensure healthy lives for them, and are obligated to hold respect for animal life”. For TNR advocates the ethical position to support the rights of domestic cats is absolute (Alley Cat Allies, 2005; Alley Cat Allies, 2011a; Alley Cat Allies, 2011b). Neither the desired outcomes of the TNR proponent nor the conservation biologist are being met concerning free-ranging cats that suffer from poor health and abuse by humans. Cat populations continue to grow, which means that small animal populations potentially suffer dramatic mortality from predation.

The positions of TNR proponents and conservation biologists represent different ends of a value orientation continuum (Vaske et al., 2001), of “enduring belief[s] that a specific mode of conduct is personally or socially preferable to an opposite or converse mode of conduct” (Rokeach, 1973:5). Vaske et al. (2001) describe a general value orientation continuum from anthropocentrism to biocentrism concerning natural resources. Here, cat welfare proponents lean toward the anthropocentric side of the continuum as domestication is a human-shaped co-evolutionary relationship with cats; moreover, pet cats are *companions to humans*. Conservation biologists lean toward biocentrism, concerned more about impacts of free ranging cats on biodiversity. In a recent study conducted in seven counties in Florida that have TNR programs, Wald, Jacobson & Levy (2013) observed this continuum in responses to survey questions concerning cat management and support of TNR programs. Members of the Audubon Society, which is an organization that is explicitly bird conservation oriented, identified more strongly with perceptions of negative impacts of cats (e.g., killing wildlife, spreading disease, and other impacts), members of TNR organizations were less perceptive of negative impacts, and randomly sampled members of the general public fell in between. An inverse pattern occurred for perceptions of positive impacts of cats (e.g., companionship and pest control). TNR supporters were more likely to consider TNR programs as effective management of free-ranging cat populations, and they were more willing to support taxation for the purpose of supporting such programs. The political ecology of ‘the free-ranging cat conundrum,’ thus, concerns an intense human environmental relationship (e.g., Ferreira et al., 2011), one that conservation scientists spend considerable time studying. However, the information produced by conservationists and the positions they hold concerning what ought to be done about free ranging cats continue to be met with resistance by proponents of TNR programs.

Contemporary research on broader impacts of science indicates that scientists struggle to communicate the merits of their research outside of the scientific community (Nadkarni & Stasch, 2013). Social scientists and environmental philosophers interested in conservation have addressed this concern by engaging local communities in conservation efforts because, though research may be scientifically valid, its implications may not be easily comprehended outside its scholarly audience (e.g., Davidson-Hunt et al., 2012; Rozzi et al., 2006). There are broader impacts of scientific conservation research relevant to pet owners who may or may not be biologically (or more broadly, scientifically) trained. Research in animal conservation is published in specialized scientific journals, such as *Conservation Biology*, *Biological Conservation*, *Animal Conservation* and similar scholarly venues. Although the scientific foundation of conservation research is critically important, we believe it has been difficult to establish a dialogue that crosses the cultural continuum between TNR proponents and conservation scientists. Without such a dialogue it has been challenging for TNR proponents and conservation biologists to reach a middle ground in which the conservation concerns and the needs of cat welfare proponents are balanced.

To encourage this dialogue we offer a contextual bridge between what we perceive are two underemphasized topics in the TNR literature. First, we provide a summary of wildcat (*Felis silvestris*) domestication, evolutionary biology, and behavioral ecology. We hold that TNR advocacy without the context provided by evolutionary biology is not well informed. The positions of conservation biologists are infused with evolutionary biology and ecology, which provides much of the reasoning that supports their biocentric ethical positions regarding free-ranging cats and small animal conservation. However, it is necessary that information regarding evolutionary biology be communicated in a manner that allows proponents of TNR to comprehend its details and its importance regarding predation caused by free-ranging cats—our review of the behavioral ecology, life-history evolution, and evolutionary biology of felids is definitively *not* for the conservation biologist (who has detailed command of this knowledge) but is instead intended to communicate to a broader audience.

In addition to this literature review, we present results of a preliminary survey of university students from a local community (the University of North Texas [UNT]) from which members knowingly or unknowingly have regular exposure to a free-ranging cat population and an active TNR program called the UNT Feral Cat Rescue Group (FCRG). Our sample of students is from core-university science classes that attract students who tend not to be versed in science and/or who have little interest in science (see Materials and Methods section). We assume these students on average are more familiar with pet ownership than they are with the goals of scientific conservation. We are interested in addressing the following research question: does presentation of a simple narrative consisting of tailored information about the conservation concerns of TNR programs change the perceptions of a generally non-scientifically trained audience? We demonstrate that such information does little to change the perceptions of the students in our sample. Given that pet owners may or may not be trained in science, conservation science might not be comprehended in such a manner that enables a dialogue between conservation biologists, TNR proponents, and members of the public who are pet owners (Peterson et al., 2012). Results of this preliminary study indicate that presentation of science-based conservation research may not be enough to initiate change in values about TNR programs, release of cats outdoors, and small animal conservation. Without a shift in values, we believe there is no middle ground for dialogue between conservationists and TNR proponents.

### Cat evolution and domestication

Exploring evolutionary history sharpens understanding of free-ranging cat predation, because it provides comprehension of the ecological role that led to the domestication of the cat and how that relationship changed over time. Domestication is a process that straddles biological and cultural spheres. Clutton-Brock (2012) describes the biological process of domestication as starting with particular members of a wild species that become accustomed to humans either through force or passively through exposure and close proximity. The relationship between the domestic cat (*Felis silvestris catus*) and its wild progenitors can be hard to tease apart due to hybridization of the different subspecies of *Felis silvestris* (Johnson & O’Brien, 1997). However, the most likely living ancestor of *F. s. catus* is *F. s. lybica*, the African wildcat (see also Randi & Ragni, 1991). Domestication might be assumed to be a function of genetic distance from the wild ancestral population; however, “[d]omestication is [also] the result of the evolution of a symbiosis” (Rindos et al., 1980:752). Rindos et al. (1980:753) frame domestication as a form of coevolution “…involving two genetically unrelated species…[that] occurs whenever the interrelationship of the organisms positively affects their potential for survival”. Therefore, domestication is founded on the *relationship* between humans and domesticates. Commonly, the human/domesticate relationship is thought of as a direct relationship; however, regarding survival, the domestic cat is somewhat unique in this regard as its association with humans can range from direct to indirect to non-existent.

It is commonly held that the cat was first domesticated in ancient Egypt, around 1900–1800 BC (Clutton-Brock, 1981; Vigne et al., 2004). More recent archaeological findings of direct human/cat interaction suggest an earlier origin in Cyprus in the Eastern Mediterranean Sea. Vigne et al. (2004) describe a cat skeleton in direct association with a human burial at Shillourokambos, a Neolithic village with an occupation from around 8000–7000 BC. This fully articulated cat skeleton was identified as *F. s. lybica*, the African wildcat, and was dated to 8300 to 8200 ^14^C years ago (roughly 7500–7200 BC). Driscoll et al. (2007) studied the geographic origins of cat domestication in the Near East by genotyping 851 short tandem repeat (STR) loci from members of *Felis silvestris*; they were able to sequence 2604 base pairs of mitochondrial DNA from 742 cats. Using neighbor-joining phylogenetic analyses, they identified six clades, or main groups, into which individual cats were distributed. They state, “[t]he composite STR genotypes of all known domestic house cats, fancy-breed cats, and feral domestic cats occurring in the wild populations all fell within a large monophyletic group (clade IV) that also included wildcats from the Near East” (Driscoll et al., 2007:521). That is, the domestic cat sits firmly in an evolutionary group (the Near East group) that derives from a common ancestor. Using a linearized tree method (see Russo, Takezaki & Nei, 1995; Lopez et al., 1997 for more details), they were able to estimate a mitochondrial gene (ND5 and ND6) sequence divergence rate of 2.24 billion base pairs per million years. This places the ancestor of Near Eastern cats (which includes *F. s. lybica* and *F. s. catus*) back 100,000 years before the discovery at Shillourokambos.

Whereas most domesticated animals have been *deliberately* bred for economic, cultural or aesthetic reasons, the domestic cat is thought to share a mutually beneficial, but low dependence relationship with humans (Clutton-Brock, 1981). The cat is also unique among domesticates because it is not an altogether social animal. Davis (1987:127 emphasis added) notes, “[c]ats are relatively solitary animals. Instead of relating to one another, they are fiercely territorial and form a strong association with their domain. A *‘domestic’ cat therefore is bonded to people’s habitation rather than to humans themselves*. In transferring odour from its scent glands by rubbing up against its owner’s legs the cat is simply including them within its territory”. Clutton-Brock (1999) and Clutton-Brock (2012) hypothesizes that this commensal relationship developed from the advent of agriculture in the Fertile Crescent where cats would have kept grain silos pest free. Near Eastern wildcats were able to occupy a new niche through commensalism with humans, from which they radiated adaptively and biogeographically.

In contemporary American society, the functional role that cats once played in early agricultural societies has been greatly reduced, though this role still exists in some rural contexts (Churcher & Lawton, 1987; Coleman & Temple, 1993; Lepczyk, Mertig & Liu, 2004; Krauze-Gryz, Gryz & Goszczynski, 2012). Human societies are increasingly urbanized (United Nations Population Fund (UNFPA), 2007; Forman, 2008; Gehrt, 2010), and cats are no longer the caretakers of our crop stores. Cats are our pets; in fact, they are the most abundant pet mammal in the United States according to the American Pet Products Association (APPA, 2013). The disjunction between the social and biological factors of early cat domestication and the contemporary environments (often urban) that pet cats now occupy is critical for understanding the context of TNR programs.

### Predatory behavior and its ontogeny

Predatory behavior is a product of life history and evolutionary biology which can be described as felid behavioral ecology. Examining predation in detail requires answering the question: why do cats hunt? One might assume that predatory behavior in cats exists solely for the purpose of food acquisition. However, Adamec (1976) has shown that predatory behavior is independent of satiation (see also Leyhausen, 1956; Leyhausen, 1979). Adamec (1976:270) describes the interaction between killing and eating prey as a set of rules that apply to certain environmental circumstances and that are generalizable to all feline predators. That is, cats kill prey when satiated as a way to maximize fitness in certain predatory contexts or as a possible contingency plan for the future (Kruuk, 1972). It is also possible that domestic cats kill by accident during play (Biben, 1979; Bradshaw, Casey & Brown, 2012).

Domesticated cats are primarily auditory hunters and have approximately 20 muscles that control the independent movement of each of their ears (Tabor, 1983; Fitzgerald & Turner, 2000). In addition, cats are visual hunters that respond to prey animals that move at particular speeds in straight paths (Fitzgerald & Turner, 2000). Domestic cats have two primary hunting strategies that are elicited by different prey encounters and environments—mobile and stationary strategies (Fitzgerald & Turner, 2000). When stalking it is advantageous for cats to be constantly on the move, but a stationary ambush strategy is more advantageous when hunting small burrowers, such as rabbits (Corbett, 1979). Mobile and stationary hunting strategies are not mutually exclusive and may be used during the same foraging expedition. Felids are largely considered nocturnal, but Fitzgerald & Turner (2000) state that domestic cats are also diurnal and suggest that this propensity may relate to their domestication and the exploitation of certain prey types that are active during the day, such as many species of birds.

Predation strategies along with preferences for particular types of prey develop early in life. Feral kittens are first introduced to prey by their mothers around 30 days after birth, which is roughly when the weaning process begins (Ewer, 1968; Baerends-van Roon & Baerends, 1979; Moelk, 1979; Deag, Manning & Lawrence, 2000). Weaning is a crucial time for development, and it causes an increase in play and predatory behavior (Caro, 1979; Caro, 1980a; Bateson & Martin, 2000; Bateson, 2000). For example, Tan & Counsilman (1985) have shown a strong correlation between early weaning and killing behavior in an experiment with laboratory mice as prey. In addition, the types of prey that mothers bring back to the den are preferentially selected by the offspring in future foraging outings (Kuo, 1930; Caro, 1980b; Bateson, 2000). Although predation strategies develop early during ontogeny, environmental contingency plays an important role in predation strategies of adult cats.

Predation behavior is phenotypically plastic, which is the propensity to exhibit “variation in the phenotype of individuals with similar genotypes due to differences in environmental factors during development” (Allendorf & Luikart, 2007:538). Not all cats are formidable predators from the start; however, as Bateson (2000:17) points out,

Despite this individual variation among young cats, however, most eventually become competent predators, albeit with different preferences and specialisations for particular types of prey…Adult predatory skills are improved by experience with prey when young, by watching the mother dealing with prey when young and, possibly, by the effects of competition between littermates in the presence of prey…Kittens that have never killed a rat, for example, can become rat-killers merely by watching another cat kill a rat…The main point here is that a given set of adult behaviour patterns—in this case predatory behaviour—is affected by several different types of experience.

Bateson goes on to describe this process in terms of the systems theory concept of equifinality where multiple possibilities can explain an observed outcome. Predatory behavior in the domestic cat is achieved through multiple routes, depending on the cat and on the context of predation.

In summary, understanding predatory behavior in cats is important for gauging the impact of TNR programs on wildlife. Predatory behavior develops at different times in the lives of different cats, and preferences for diverse predation strategies and types of prey vary by cat and context. It is clear that despite their relationship with humans, in terms of their evolutionary biology cats are predators. Thus, free-ranging cat predation on small mammals, reptiles, amphibians, and birds is of considerable conservation concern. Cat domestication started as a relationship based on its behavioral ecology, much like the domestication of the dog did. However, the social nature of dogs is much more amenable to the shift away from *domesticate as foraging partner* to *contemporary companion pets* (Shultz & Dunbar, 2010). As human relations with cats have changed toward companionship in contemporary society, however, cats (particularly free-ranging ones) have remained closely attuned to their evolutionary biology, that which made them an efficient partner to humans in the past, *their solitary predatory nature*.

Not all pets are created equal in terms of predatory ecology (e.g., dogs and cats are fundamentally different in terms of evolutionary biology and behavioral ecology), and this perspective has been largely ignored by TNR proponents. In contrast, conservation scientists take the predator ecology of cats for granted and may overlook the importance of cats as pets. We make the relatively safe assumption that members of the public without scientific training in biology are unlikely to be informed about the predator ecology of cats. In addition, we conjecture that most members of the public, including a substantial segment of society who are not trained biologists, are very familiar with cats as pets. In order to learn more about how non-scientists interact with free-ranging cats, how aware they are of a local TNR program, and how they respond to information on cat ecology and small animal conservation, we conducted a pilot survey among members of a local community who are consistently in close proximity with free-ranging cats and a TNR program, students (primarily non-science majors) at the University of North Texas.

## Materials & Methods

We are interested in addressing the following research question: does presentation of a simple narrative consisting of tailored information about the conservation concerns of TNR programs change the perceptions of a generally non-scientifically trained audience? Secondarily, we are interested in gauging the familiarity of this audience with a local TNR program (as prescribed by Loyd & Miller, 2010).

We approached this question by conducting a simple survey using a convenient, judgmental sample of students in particular core-science classes at the University of North Texas. Students at UNT are knowingly or unknowingly regularly in close proximity to cat houses and feeding stations operated by the UNT FCRG. We selected core classes that draw students from all colleges and most departments at UNT representing a high diversity of majors. Students must choose from natural and physical science core classes to meet their degree requirements. Our sample is not representative of the general public and may not represent the UNT student body as a whole; however, characteristics of the classes we sampled lead us to conclude that this sample represents an audience dominated by individuals without interests in science or who are not scientifically trained.

Earth Science (physical science option of the UNT core) and Archaeological Science (natural science option of the UNT core) were judgmentally sampled for important reasons; first, the courses typically draw those students who exhibit relatively low scientific literacy or interest compared to those drawn to biology, chemistry, physics, and astronomy core science classes (the other available core options). Second, S Wolverton has taught both classes for several years and J Dombrosky is a TA for Archaeological Science, thus we are certain no information on domestication of cats or on biological conservation had been covered in either class prior to administering the survey. This does not preclude that students had little or no knowledge of TNR and/or small animal conservation prior to entering the class, the gauging of which represents one goal of the survey. Third, our response rate was high due to choosing a captive audience and administering the survey face to face; we faced essentially no problem of non-response bias (Vaske, 2008).

We crafted two narratives, one to introduce TNR programs and to gauge students’ familiarity with, and impressions of, such programs. The presentation of this first narrative was followed by a short survey. The second narrative summarized the animal conservation concerns related to TNR programs, which was utilized to determine if student perceptions of TNR programs shift once presented with tailored information. Crafting of the conservation narrative required portraying the position of conservation biologists, which included using terms common in conservation discourse, such as “invasive”, “predator” and “threat”. Many elements of our survey design follow recommendations by Dillman (2007) for mail and web-based surveys. These include, assessing the interest and validity of the questions we ask, keeping question style and structure succinct and simple (e.g., avoiding compound sentence questions), aspects of question presentation style, ordering of questions as well as magnitude and direction of Likert scale response items. However, our survey design aligns more closely with a tailored marketing analysis used to gauge the impact of crafted narrative on respondent perceptions, which is more common in health education research (Campbell et al., 1999; Brug, Oenema & Campbell, 2003; Kreuter & Wray, 2003).

Our approach introduced a common problem in face-to-face survey administration, that of interviewer bias (Podsakoff et al., 2003). We controlled for this in three ways. We crafted neutral language to describe TNR because we are scientists with backgrounds in ecology who lean toward biocentrism. Terms such as “abandon”, “feral” and “killing” were balanced with “avoid”, “provide” and “humane”. Second, after obtaining informed consent, we read the TNR description, paying careful attention to tone of voice so as not to portray TNR programs negatively, after which we offered a short survey. The TNR narrative was displayed via overhead projection so that students could refer back to it while taking the survey. Third, we placed the TNR description and survey response to it prior to exposure to narrative explicating conservation concerns. We sampled students from Earth Science on Thursday March 15, 2012 at approximately 3:00 pm (GEOG 1710; *n* = 100) and Archaeological Science on Tuesday March 27, 2012 at approximately 11:10 am (ARCH 2800; *n* = 178). Data on seven demographic characteristics were collected: age, gender, major(s), childhood in an urban or rural area, type of current residence, history of cat ownership, and how many cats are cared for currently (Table 1). The following prompt was then displayed via overheard projection and read aloud:

Cats that are not pets are known as feral cats. Trap–Neuter–Return (TNR) is a national program committed to the humane management of feral cats. It is implemented in urban areas as well as on many campuses, including UNT. In urban areas it is common for people to abandon cats. At UNT, the program operates by providing small green houses for shelter and also provides food for these animals. The houses are often checked for feral cat occupancy. If a new feral cat is found, it is trapped, then neutered or spayed (a surgery making the animal incapable of breeding), and finally released to the area where it was trapped. The point of the program is to humanely minimize or halt feral cat population growth. However, in an urban setting, including college campuses, cats are constantly abandoned, which provides a continual supply of feral cats. Ideally, the program attempts to avoid the killing of these animals.

Respondents were then asked to fill out the first portion of the survey consisting of five questions about their opinion of the TNR program, three of which used a Likert scale (the full survey is provided in Supplemental Information). After completing the first section, respondents were shown and read a second prompt:

Cats are an invasive species. Therefore, within urban and rural areas cats are not a naturally occurring species. Other native species have not evolved with the domestic cat. Cats are efficient predators; they hunt even when they are not hungry. Research has shown that cats are a danger to wildlife, including native and migratory bird species. Birds help maintain insect populations and disperse seeds. Feral cat predation poses a potential threat to wildlife. One solution is to keep domestic cats indoors. Euthanasia may be an appropriate alternative to *feral* cat management (emphasis in the original).

Respondents were then asked to fill out a second portion of the survey consisting of 4 questions, also using a Likert scale (see Supplemental Information).

**Table 1:** Demographic descriptive statistics of surveyed students.

		Total	Males	Females
**Age**	Average	21.2	22	20
	Minimum	18	18	18
	Median	20	20	19
	Maximum	44	44	36
**Have ever had a cat**	Yes	162 (58.91)	62 (57.41)	100 (59.88)
	No	113 (41.09)	46 (42.59)	67 (40.12)
**Currently care for cat**		91 (33.10)	32 (29.36)	59 (35.54)
**Number of cats cared for by caregivers**	Average	1.82	1.75	1.86
	Minimum	1	1	1
	Median	1	1	1
	Maximum	10	10	6
**Residency**	Apartment	84 (30.55)	37 (33.94)	47 (28.31)
	Dorm	111 (40.36)	31 (28.44)	79 (47.59)
	House	80 (29.09)	41 (37.62)	40 (24.10)
**Upbringing**	Urban	189 (68.73)	71 (65.14)	117 (70.48)
	Rural	79 (28.73)	36 (33.03)	44 (26.51)
	Both^*^	7 (2.54)	2 (1.83)	5 (3.01)

**Notes.**

* This group was not given as a choice in the survey but was written in by respondents. () represent percentages.

To understand how much students know about the TNR program implemented on campus and to ascertain the impact of tailored conservation-oriented information on perception, Wilcoxon Signed-Rank tests were run for responses to “I support the TNR program” in parts one and two of the survey. Comparisons were made for all respondents, males and females separately, and respondents who both knew and did not know about TNR programs to determine if established knowledge about TNR affected shifts in opinion. In addition, we separated the sample by class to test whether or not students with a presumably greater knowledge in basic ecology, geography, and earth processes in Earth Science responded differently than students without such basic knowledge in Archaeological Science. The Wilcoxon test assesses if and how (+/−) responses significantly change (e.g., if perception significantly changes) between the first and second parts of the survey.

## Results and Discussion

Respondents who left a demographic question blank, any of the questions blank on the first part of the survey, or questions 1 or 2 blank on the second part of the survey were excluded from the analysis (*n* = 3). The original sample size (*n* = 278) was corrected (*n* = 275).

Demographic characteristics of respondents in the sample are provided in Table 1. Of the sampled population, 65% (*n* = 179) did not know about the existence of TNR programs, and 35% (*n* = 96) did know about them. Of those who knew about TNR programs, only 21% (*n* = 20) knew that the UNT FCRG operated on campus. Overall, only 7% of the sampled population knew that the FCRG existed. Despite the fact that FCRG has been active on the UNT campus for approximately 15 years and that feral cat houses and feeding facilities are visible in many areas of campus, greater than 90% of the respondents were unaware of the program.

To determine if perceptions of TNR programs change with exposure to our tailored conservation narrative Wilcoxon Signed-Rank tests were run using five response categories (1 = Strongly Disagree, 2 = Disagree, 3 = Neutral, 4 = Agree, and 5 = Strongly Agree) from questions five on the first part of the survey and question two on the second part of the survey (i.e., “I support the TNR program”). Table 2 provides the results of six sets of Wilcoxon Signed-Rank tests, which were run for the sample as a whole, the sample separated into males and females, the sample grouped into males and females who did and did not know about TNR programs, and for Archaeological Science and Earth Science classes. Significant results were obtained for every test but one, males who knew about TNR programs. For the other groups, exposure to tailored conservation information led to a less favorable impression of TNR programs. Effect size is reported to assess the magnitudes of change for each significant Wilcoxon Signed-Rank test (Cohen, 1988); most of the significant outcomes exhibit low effect size.

**Table 2:** Wilcoxon signed-rank statistics of perception shifts between parts 1 and 2 of the survey.

Statistic	Whole sample	Gender		Know about TNR		Did not know about TNR		Archaeological Science	Earth Science
		Male	Female	Male	Female	Male	Female		
*z* ^*^	−5.57	−2.09	−5.41	−.690^**^	−3.53	−2.13	−4.22	−3.57	−4.41
*p*	.001	.040	.001	.490	.001	.034	.001	.001	.001
Effect size (*r*)	.34	.20	.42	.11	.48	.26	.40	.27	.44

**Notes.**

* Negative *z* scores refer to direction of perception shift.

** Indicates only non-significant value.

We consistently observed significant shifts in how students responded to our questions about the UNT FCRG program and TNR programs. Tailored information on small animal conservation and the impacts of free-ranging cats had an impact on student perceptions. In general, students became less supportive of TNR programs after reacting to information on free-ranging cats as small animal predators. This result supports Wald, Jacobson & Levy’s (2013) conclusion that stakeholders who know very little about free-ranging cats, TNR, and conservation (65% of the students here) may be “susceptible to form effects, due to biased framing or terminology”, such as the terms “invasive” and “danger” in our conservation narrative. However, *despite that there is a significant response* to information on the impact of cats as predators, the magnitude of *the shift in student perception is not large*. That is, effect size is weak to moderate at best when the student sample is aggregated as a whole, which generally echoes the significant but low effect-size results of Wald, Jacobson & Levy (2013). However, when the student sample is separated by course, effect size of the response in Earth Science students is markedly larger than in Archaeological Science students (Table 3), which we return to below.

**Table 3:** Ordinal scale perception shifts in Archaeological Science and Earth Science students.

	Archaeological science		Earth science	
Change in rank	Count	%	Count	%
Negative	35	20	30	30
Positive	10	6	4	4
None	131	74	65	66
Total	176	100	99	100

This study is preliminary and results are based on a convenient, judgmental sample; however, the small effect of new information on student perceptions is not surprising and may point toward a fundamental problem in the free-ranging cat debate. Proponents of TNR programs and those of small animal conservation appear to be talking past one another from endpoints on the value orientation continuum between anthropocentrism and biocentrism (Vaske & Donnelly, 1999). If so, proponents on both sides assume that disparate sources of information will influence values and shift perspectives to either promote TNR programs or to instill conservation values. It is becoming increasingly clear in the biocultural conservation literature, however, that information-based approaches to conservation may simply be expedient with similar effects to what has been termed ad hoc conservation (Pressey & Tully, 1994; Mills et al., 2012; Saslis-Lagoudakis & Clarke, 2013). Expedient conservation is marginally effective at best and ineffective at worst. A fuller context of cats-as-pets and the evolutionary biology of cats-as-predators, while implicit in the biocentric perspectives of conservation biologists, is not easy to communicate to non-biologists. Contemporary scholars of environmental ethics and biocultural conservation recognize that values related to knowledge underlie individual decisions, such as whether or not to allow pet cats outdoors, whether or not to support TNR programs, or whether or not to support local conservation efforts (Colding & Folke, 2001; Lertzman, 2009; Rozzi, 1999; Rozzi et al., 2006; Vandebroek et al., 2011; Wyndham, 2009).

Conservation biology that incorporates local knowledge, values, and perceptions emphasizes the role that “ecological understanding” plays in behavior related to human–environment interactions (Turner & Berkes, 2006:497; see also Rozzi, 1999; Rozzi et al., 2006). Such ecological understanding, which comprises beliefs and practices that relate to values developed through direct encounter with the outdoor environment, may not necessarily be a common experience for members of the general public including cat owners and proponents of TNR programs in the US (and in similar Western countries with similar pet-ownership practices). It is more likely that conservation biologists embed ecological understanding within their biocentric value orientation than do the average American pet owners, average proponents of TNR programs, or even members of the public who do not own pets. In addition to their formal training in biology, conservation biologists regularly and directly encounter the outdoor environment in the field, so a greater level of ecological understanding is to be expected.

As a result, we suggest that the free-ranging cat/small animal conservation problem cannot be solved with new information alone. What must change are the social values that underlie pet ownership, abandonment, and environmental ethics, holistically. This type of focus on environmental ethics has led to innovative conservation initiatives, such as participatory conservation, environmental co-management, and environmental education (Berkes, 2007; Davidson-Hunt et al., 2012; Mills et al., 2012; Müller & Guimbo, 2010; Mulrennan, Mark & Scott, 2012; Ostrom, 2007; Rozzi et al., 2006). Although, community based approaches to conservation are not without their problems (Mulrennan, Mark & Scott, 2012), it will take this type of initiative to change the social context of free-ranging cat populations, which includes value orientations about pet ownership and abandonment.

Imagine a more environmentally knowledgeable citizenry in which the context of ecology, that of connections among humans and environment, are more embedded in values, perceptions, and decisions (e.g., Turner & Berkes, 2006). The problem is not one of information (e.g., Lepczyk, 2005); it is one of environmental values. A pet owner with greater ecological understanding is more likely to monitor her/his cat’s free-ranging behavior and predatory impacts than one who is not ecologically knowledgeable. Inasmuch as Florida residents who are members of the Audubon Society have greater ecological understanding, the previously mentioned results from the survey study by Wald, Jacobson & Levy (2013) support this argument.

Despite the small scale and simplicity of our study, we believe there is variability in ecological understanding playing out in this sample of UNT students. The different effect size in responses of Earth Science and Archaeological Science students to tailored conservation information is reflected in the percent of students in each class who responded positively or negatively to TNR programs after receiving the information (Table 3). This may represent an example of Earth Science students being moderately more ecologically knowledgeable than students of Archaeological Science, and thus may represent the type of environmental education that can influence perceptions of conservation information. The Earth Science students surveyed here were taught basic ecology, core ecosystem concepts, and geographic concepts that connect people to environments early in the course. In contrast, Archaeological Science focuses first on the precepts of science and scientific literacy as well as the general framework for studying archaeology. Although Earth Science students were taught basic concepts in ecology and geography, these concepts were not explicitly linked to conservation science (which takes place later in the course, well after the survey was administered). In particular, there was no mention of bird conservation or the free-ranging cat debate until after the survey took place. In most aspects, the student bodies of the courses are similar. There is no reason to expect that students in Archaeological Science were any less able to understand the fundamental concepts of TNR programs or the conservation implications of free-ranging cats. We suspect that Earth Science students were simply more knowledgeable of ecology, human–environment interactions, and earth processes because of the concepts they received early in the course, which may have prepared them to think more openly about conservation. Confirmation of our position would require a more in-depth study of these types of classes; here we simply raise it as a potential explanation for the difference we observed.

Abandonment of pet cats by students is likely to be an important contribution to the free-ranging cat populations on campus at UNT (Hughes & Slater, 2002). To reduce cat abandonment, which would ultimately serve the goals of conservationists and TNR proponents, our results indicate (in addition to those of Wald & Jacobson, 2013; Wald, Jacobson & Levy, 2013) that an information-based campaign concerning the small animal conservation risks of releasing free-ranging cats would be only marginally effective. We propose that a more effective approach would be to adopt the UNT-FCRG and the campus free-ranging cat population as an example of a local conservation issue of concern in required core science classes, such as introductory survey courses in biology, environmental science, political science, philosophy, anthropology, and earth science in which the value orientation continuum between TNR proponents and conservationists can be explored and discussed. Although we cannot fully support this course of action based on the results of this preliminary study, our position is also supported by many studies in biocultural conservation and environmental education.

## Conclusion

Reduction of free-ranging cat populations can only occur if conservationists work to shift the context of pet ownership, abandonment, and coexistence with free-ranging cat populations. This should start with an explicit acknowledgement that TNR programs are part of the solution for controlling free-ranging cat populations rather than an entry point for an antagonistic debate. As a case study, the free-ranging cat debate is a prime example of what Nabhan (2013) has termed “autobiology”, the ethnobiology of ourselves (Westerners, Euro-Americans, those in societies with cats as pets in this case). Autobiology is simply ethnobiology within one’s own culture, and contemporary ethnobiology is often defined as the study of human interactions with biota in environments (Anderson, 2011; Wolverton, 2013). The free-ranging cat debate represents an opportunity to teach evolution, ethnobiology, and ethics in the same setting for the benefit of increasing ecological understanding (*sensu* Turner & Berkes, 2006). In order to achieve the goals of integrating the debate into environmental education in this manner, however, conservation biologists must recognize that the impact on values and conservation may be general, somewhat vague, and immeasurable on short time scales that are common metrics of successful conservation. That is, conservation biologists must bank on shifts in values through trusting the outcomes of environmental education rather than immediate response through forceful debate or ad hoc conservation.

Cats as pets can be contextualized into the evolutionary history of domestication. Doing so provides a basis for comprehending both the value of cats as companions and pets but also the conservation risks of releasing them to the outdoors. There are very clear reasons to expect that free-ranging cats will act as predators of small animals that are related to evolutionary biology, life history evolution, behavioral ecology, and the co-evolutionary process of domestication. However, an information-based approach that simply presents the evolutionary biology of cats as predators is unlikely to dramatically influence the values upon which pet owners base their decisions to own cats and to release them outdoors. We believe that a reason for the ineffectiveness of an information-based approach is that the context of pet ownership in terms of environmental ethics does not necessarily shift due to new information provided in what may turn towards an antagonistic debate. The relevance of information related to the perspectives of conservation biologists on any single issue is more likely to make a difference in the context of environmental education that engages the members of the local community that are involved in decision making.

## Supplemental Information

10.7717/peerj.312/supp-1 Supplemental Information 1 UNT IRB Approval letter

10.7717/peerj.312/supp-2 Supplemental Information 2 Original survey administered to UNT students
