# Supplementary material for: TNR and conservation on a university campus: a political ecological perspective
Source: PeerJ. 2014 Mar 18;2:e312. doi: 10.7717/peerj.312 (PMC3970807; doi:10.7717/peerj.312)
Supplement: Supplemental Information 2 [file peerj-02-312-s002.docx]

**Part One**

**Please answer the following general questions about yourself:**

1. **Age**_____
2. **Gender:**

a. female b. male

1. **Major(s)**__________________________________
2. **What kind of area were you raised in?**

a. urban area b. rural area

1. **Where do you live?**

a. Dorm b. Apartment c. House

1. **Have you ever had a pet cat?**

a. Yes b. No

1. **How many cats do you care for?** __________

**Please answer the following questions after a brief introduction to the TNR program (only circle one answer for each question or statement):**

1. **Did you know these kinds of programs existed?**

a. Yes b. No

1. **Did you know this program existed at UNT?**

a. Yes b. No

1. **It is important to me that feral cats are treated humanely.**

| Not at All Important | Slightly Important | Moderately Important | Very Important | Extremely Important |
| --- | --- | --- | --- | --- |

1. **Feral cats have the right to live.**

| Strongly Disagree | Disagree | Neutral | Agree | Strongly Agree |
| --- | --- | --- | --- | --- |

1. **I support the TNR program.**

| Strongly Disagree | Disagree | Neutral | Agree | Strongly Agree |
| --- | --- | --- | --- | --- |

**Do Not Proceed Until the Next Prompt**

**Part Two**

**Please answer the following questions after an introduction to a biological perspective (only circle one answer for each question):**

1. **How acceptable is it to euthanize feral cats to protect wildlife?**

| Not at All Acceptable | Slightly Acceptable | Moderately Acceptable | Very Acceptable | Extremely Acceptable |
| --- | --- | --- | --- | --- |

1. **I support the TNR program.**

| Strongly Disagree | Disagree | Neutral | Agree | Strongly Agree |
| --- | --- | --- | --- | --- |

1. **It is unethical to support one species’ existence over another.**

| Not at All Ethical | Slightly Ethical | Moderately Ethical | Very Ethical | Extremely Ethical |
| --- | --- | --- | --- | --- |

1. **It is better to say that all species have the right to *try* to live instead of all species have the right to live.**

| Strongly Disagree | Disagree | Neutral | Agree | Strongly Agree |
| --- | --- | --- | --- | --- |
