# Supplementary material for: TNR and conservation on a university campus: a political ecological perspective
Source: PeerJ. 2014 Mar 18;2:e312. doi: 10.7717/peerj.312 (PMC3970807; doi:10.7717/peerj.312)
Supplement: Supplemental Information 1 [file peerj-02-312-s001.pdf]

UNT™
UNIVERSITY OF
NORTH TEXAS
Discover the power of ideas.

OFFICE OF THE VICE PRESIDENT FOR RESEARCH AND ECONOMIC DEVELOPMENT
Research Services

March 6, 2012

Dr. Steve Wolverton
Department of Geography
University of North Texas
RE: Human Subjects Application No. 12-066

Dear Dr. Wolverton:

In accordance with 45 CFR Part 46 Section 46.101, your study titled "Student Perception of Feral Cats and their Effects on Campus Wildlife" has been determined to qualify for an exemption from further review by the UNT Institutional Review Board (IRB).

Enclosed is the consent document with stamped IRB approval. Please copy and **use this form only** for your study subjects.

No changes may be made to your study's procedures or forms without prior written approval from the UNT IRB. Please contact Jordan Harmon, Research Compliance Analyst, ext. 3940, if you wish to make any such changes. Any changes to your procedures or forms after 3 years will require completion of a new IRB application.

We wish you success with your study.

Sincerely,

Patricia L. Kaminski, Ph.D.
Associate Professor
Chair, Institutional Review Board

PK:jh

1155 Union Circle #305250 | Denton, Texas 76203-5017 | TEL 940.565.3940 | FAX 940.565.4277
TTY 940.369.8652 | http://research.unt.edu

Informed Consent Notice

Before agreeing to participate in this research study, it is important that you read and understand the following explanation of the purpose, benefits and risks of the study and how it will be conducted.

Title of Study: Student Perception of Feral Cats and Their Effects on Campus Wildlife

Student Investigator: Jonathan Dombrosky, University of North Texas (UNT) Department of Anthropology. **Supervising Investigator**: Dr. Steve Wolverton

Purpose of the Study: You are being asked to participate in a research study which involves filling out a survey so we can gauge your opinion on an environmental program on campus.

Study Procedures: You will be asked to fill out a demographics portion of a survey, and then you will be asked to listen/read two prompts and complete a one page questionnaire for each prompt in regards to your opinion that will take about 15 minutes of your time.

Foreseeable Risks: No foreseeable risks are involved in this study

Benefits to the Subjects or Others: This study is not expected to be of any direct benefit to you, but we hope to learn more about student opinion about environmental programs at UNT, and infer information about collegiate student populations nationwide. We hope to understand student knowledge, awareness, and opinion of their campus viewed from an ecological perspective.

Compensation for Participants: None

Procedures for Maintaining Confidentiality of Research Records: We are not collecting personally identifiable information. The confidentiality of your individual information will be maintained in any publications or presentations regarding this study.

Questions about the Study: If you have any questions about the study, you may contact *Jonathan Dombrosky* at *jwdombrosky@gmail.com* or *Dr. Steve Wolverton* at *wolverton@unt.edu.*

Review for the Protection of Participants: This research study has been reviewed and approved by the UNT Institutional Review Board (IRB). The UNT IRB can be contacted at (940) 565-3940 with any questions regarding the rights of research subjects.

Research Participants' Rights:

Your participation in the survey confirms that you have read all of the above and that you agree to all of the following:

- *Jonathan Dombrosky* has explained the study to you and you have had an opportunity to contact him/her with any questions about the study. You have been informed of the possible benefits and the potential risks of the study.
- You understand that you do not have to take part in this study, and your refusal to participate or your decision to withdraw will involve no penalty or loss of rights or benefits. The study personnel may choose to stop your participation at any time.
- *Your decision whether to participate or to withdraw from the study will have no effect on your grade or standing in this course.*
- You understand why the study is being conducted and how it will be performed.
- You understand your rights as a research participant and you voluntarily consent to participate in this study.
- You understand you may print a copy of this form for your records.

APPROVED BY THE UNT IRB
DATE: 3-6-12
